# Book Review of Handbook of Elemental Speciation Techniques and Methodology

**DOI:** 10.1155/S1463924603000257

**Published:** 2003

**Authors:** Peter B. Stockwell


Journal of Automated Methods & Management in Chemistry
Vol. 25, No. 6 (November–December 2003) p. 149

### Book Review

### Handbook of Elemental Speciation Techniques and Methodology

Edited by Chief Rita Cornelis

Chichester: Wiley. ISBN 0-471-49214-0

By chance, two works on speciation arrived on my desk at almost the same time. The first being the present book, the second being an 'A' page article published in *Analytical Chemistry* by Chris Le and co-workers [1].

Speciation analysis is a complex task and a reference handbook on relevant techniques and methodology has been required by all those with an interest in the requirements for speciation analysis.

The Preface of the present book quotes:

> These expectations are now fulfilled by the *Handbook of Elemental Speciation*. ...The first volume brings together a collection of chapters covering comprehensively different aspects of procedures for speciation analysis at the different levels starting from sample collection and storage, through sample preparation approaches to render the species chromatographable, principles of separation techniques used in speciation analysis, to the element-specific detection.

Does the book deliver what it suggests? The answer is only in part! It is a welcome contribution to the literature, but it only deals with a section of the available literature. It is directly influenced by the areas of work covered by the contributors and does not widen the scope outside their areas of interest. The A-page article, on the other hand, draws a comparison of the predominant techniques described in the book with atomic fluorescence spectroscopy speciation profiles. Whereas the *Handbook* only pays passing reference to the atomic fluorescence spectroscopy technique, it is in stark contrast to the available literature. While it offers some introduction to the techniques of speciation, it provides only references to the techniques used by the syndicate of authors. The ICP-MS measurement technique does offer good detection levels, but only at costs that cannot be borne by most routine laboratories or laboratories in Third World countries.

The most difficult area of speciation remains the provision of a truly representative sample and the ability to transfer this to a suitable measurement technique whilst ensuring that the speciation profile remains intact. For laboratories starting this difficult technique, this book will provide a useful insight into the problems. To gain real experience, they will better be served by working closely with groups with proven expertise in analysing samples effectively and reliably.

*Peter B. Stockwell*

### Reference

1. Le, C. *et al.*, *Analytical Chemistry*, **76** (2004), A27.

